# Reference intervals of complete blood count and coagulation tests in Vietnamese pregnant women

**DOI:** 10.1186/s12884-023-06106-2

**Published:** 2023-11-11

**Authors:** Huan Nguyen Pham, Nghiem Xuan Huynh, Phuc Nguyen Huu Pham, Dung Ngoc Yen Dang, Long Thang Cao, Diem Minh Huynh, Hau Thi Thu Thoi, Oanh Hoang Le, Suzanne Monivong Cheanh Beaupha

**Affiliations:** 1 Hung Vuong Hospital, Ho Chi Minh City, Vietnam; 2 University of Medicine and Pharmacy at Ho Chi Minh City, Ho Chi Minh City, Vietnam; 3 Cho Ray Hospital, Ho Chi Minh City, Vietnam

**Keywords:** Reference interval, Pregnancy, Vietnamese, Complete blood count, Coagulation

## Abstract

**Background:**

Pregnancy has major effects that make hematology parameters outside of normal reference ranges. Therefore, we conducted this study to establish reference intervals for Vietnamese pregnant women.

**Methods:**

From June 2023 to Augst 2023, blood samples from 879 eligible pregnant women were run on DxH 900 hematology analyzer and ACL TOP 550 coagulation analyzer. The tested parameters are prothrombin time (PT), activated partial thromboplastin time (APTT), fibrinogen (FIB), white blood cell (WBC) and its differentials (neutrophils, lymphocytes, monocytes, eosinophils and basophils), red blood cell (RBC), hemoglobin (HGB), hematocrit (HCT), mean corpuscular volume (MCV), mean corpuscular hemoglobin (MCH), mean corpuscular hemoglobin concentration (MCHC), RBC distribution width (RDW), RBC distribution width standard deviation (RDW-SD), platelet count (PLT), mean platelet volume (MPV). A non-parametric method was used to establish the 2.5^th^ and 97.5^th^ percentile reference intervals.

**Results:**

PT, APTT decrease but fibrinogen increases during pregnancy. Physiological adaptations of pregnancy result in a decrease in RBC count, but an increase in WBC count and no changes in platelet count. The reference intervals for PT (seconds), APTT (seconds), fibrinogen (mg/dL), in the first trimester were 10.30–12.88, 25.40–35.46, 280.28–559.00, in the second trimester were 9.80–11.66, 24.05–33.23, 347.75–593.35, in the third trimester were 9.60–11.40, 23.40–31.80, 330.28–628.56, respectively. The reference intervals for main hematology parameters which are WBC (× 10^9^/L), RBC (× 10^12^/L), HGB (g/dL), HCT (%), PLT (× 10^9^/L) in the first trimester were 6.33–15.24, 3.73–5.32, 10.33–13.95, 32.22–42.29, 169.66–413.88, in the second trimester were 6.99–15.55, 3.33–4.98, 9.71–13.17, 30.26–40.07, 172.34–372.19, in the third trimester were 6.22–14.14, 3.54–4.98, 9.80–13.97, 31.11–42.70, 151.30–417.14, respectively.

**Conclusions:**

Most established referenced intervals from each trimester differ from other trimesters. These trimester-specific reference ranges for Vietnamese pregnant women will aid clinicians in entepreting parameters and help other laboratories adopt these ranges after validating.

**Trial registration:**

This study is registered at www.clinicaltrials.gov as NCT05929326.

## Introduction

Reference interval is defined as the interval between and including two numbers, an upper and lower reference limit, which are usually the 2.5^th^ percentile to 97.5^th^ percentile of a normal population. Therefore, it is common to interpret results as abnormality based on normal reference intervals in pregnant women due to their biophysiological changes [1]. As a result, it is crucial to define normal reference intervals for pregnant women. Complete blood count and coagulation tests such as activated partial thromboplastin time (APTT), prothrombin time (PT), and fibrinogen are of a routine hematology panel. There have been many studies conducted on Asian pregnant women [2–4], but none was conducted in Vietnam. Therefore, we conduct this study to determine trimester specific reference intervals for healthy pregnant women.

## Materials and methods

### Patient selection

From June 2023 to August 2023, pregnant women visiting obstetrics clinics at Hung Vuong Hospital are selected if eligible after history taking and clinical examination. Inclusion criteria were singleton pregnancy. Exclusion criteria were hypertension, diabetes, pre-eclampsia, gestational diabetes, hemoglobinopathy, current infection, positive screening for *treponema pallidum*, hepatitis B virus, HIV, usage of anticoagulant drug. To establish reference intervals using non-parametric method, the minimum sample size needed is 120, according to CLSI EP28-A3c [5], because of the 90% confidence interval reported for the 2.5^th^ percentile. However, Sample Sizes for Clinical, Laboratory and Epidemiology Studies [6] suggested a calculation for larger minimum required sample size ${N}_{Rank}=\eta \sqrt{3}\times {\left[\frac{{Z}_{1-\gamma /2}}{{{\rho }_{plan}\times Z}_{1-\alpha /2}}\right]}^{2}$ where $\eta =\frac{\sqrt{\left(\gamma /2\right)\left[1-\left(\gamma /2\right)\right]}}{{\phi }_{1-\gamma /2}} =2.11$ (${\phi }_{1-\gamma /2}= \frac{1}{\sqrt{2\pi }}{e}^{\frac{{Z}_{1-\gamma /2}^{2}}{-2}}$) with α = 0.05, γ = 0.1. ρ_plan_ is the margin of error, which is the estimate of the percentage that the width of the confidence interval of the reference limits is of the width of the reference interval. The recommended ρ_plan_ is 10%. From the formula above, the calculated sample size required is 258 patients. Because we want to establish trimester specific reference intervals, the total combined number of each trimester is at least 774 patients.

### Samples collection

Venous blood samples were collected in trisodium citrate 3.2% and K_2_EDTA plastic whole blood tube and then immediately analyzed.

### Instruments and analysis

Coagulation assays, which comprises of Activated partial thromboplastin time (APTT), prothrombin time (PT), fibrinogen (FIB), was performed on ACL TOP 550 automated coagulation analyzer (Werfen, Barcelona, Spain) and complete blood count, including White blood cell (WBC) counts and percentage and absolute count of different leukocytes (neutrophils, lymphocytes, monocytes, eosinophils and basophils), red blood cell (RBC), hemoglobin (HGB), hematocrit (HCT), mean corpuscular volume (MCV), mean corpuscular hemoglobin (MCH), mean corpuscular hemoglobin concentration (MCHC), RBC distribution width (RDW), RBC distribution width standard deviation (RDW-SD), platelet count (PLT), mean platelet volume (MPV), was performed on DxH 900 automated hematology analyzer (Beckman Coulter, CA, USA).

All instruments were maintained and calibrated according to the manufacturers’ instructions. Two levels of controls were done on ACL TOP 550 analyzer every eight-hour shift and three levels of controls were run DxH 900 once a day. The average analytical precision of PT, APTT, Fibrinogen across two levels of controls is 2.00%, 1.26% and 4.83%, respectively. The average analytical precision of WBC, RBC, HGB, HCT, PLT across three levels of controls is 1.45%, 0.90%, 0.60%, 1.07% and 1.46%, respectively.

### Statistics

Patients were divided into three groups, which are first, second, and third trimester. CLSI EP28-A3c recommended Dixon’s D/R ratio outlier test to exclude outliers, where D is the absolute difference between the largest (or smallest) and the second largest (or smallest) observation, and R is the range of all observations. If D is equal to or greater than one-third of R, that observation is considered an outlier and is deleted. After outliers were deleted, the remaining data was tested again for additional outliers until there were no more outliers. A non-parametric method, recommended to establish reference intervals by CLSI EP28-A3c, was used to determine the lower reference limit 2.5^th^ percentile and upper reference limit 97.5^th^ percentile for each group. Each reference limit was reported along with its 90^th^ confidence interval. Groups were then compared using normal approximation with continuous correction Mann–Whitney U. A two-tailed *P*-value < 0.05 was considered significant. Statistical analysis was performed using NCSS statistical software (Utah, USA) version 2023.

## Results

A total of 879 patients were enrolled but 12 patients’ samples failed to run, leaving 867 patients. Out of 867 patients, due to unqualified quality, 837 samples were eligible for complete blood count test and 836 samples for coagulation tests. The characteristics of the women measured before being enrolled were described in Table 1. Overall, most women are in the age of 25 to 33. In the first trimester, one outlier was detected in APTT parameter, one outlier in monocyte parameter and one outlier in basophil parameter. In the second trimester, two outliers were detected in RDW-SD parameter. In the third trimester, only one outlier was detected in eosinophil parameter. Tables 2 and 3 illustrate the reference intervals, for complete blood count test and coagulation tests, respectively, along with 90^th^ confidence intervals for upper and lower limits. Table 4 describes verification study for transference of reference interval from two studies on Asian pregnant women.

**Table 1:** Characteristics of pregnant women in this study

Characteristics	First trimester (*n* = 305)			Second trimester (*n* = 276)			Third trimester (*n* = 286)		
	**Median**	**25**^**th**^** percentile**	**75**^**th**^** percentile**	**Median**	**25**^**th**^** percentile**	**75**^**th**^** percentile**	**Median**	**25**^**th**^** percentile**	**75**^**th**^** percentile**
**Age (years)**	28	25	33	28	25	32	29	25	33
**Systolic pressure (mmHg)**	111	108	120	114	110	120	116	110	120
**Diastolic pressure (mmHg)**	70	64	73	70	64	76	70	62	75
**Gestational age (weeks)**	11	9	12	25	24	26	37	36	37
**Weight (kg)**	57	50	64	59.5	54	66	60	53.4	67
**Height (cm)**	157	153	160	157	153	160	157	152	160

**Table 2:** Reference intervals for complete blood count parameters during pregnancy

	**First trimester (*n* = 287)**			**Second trimester (*n* = 273)**			**Third Trimester (*n* = 277)**		
Parameter	**2.5**^**th**^** (90% CI)**	**97.5**^**th**^** (90% CI)**	***P***_1_^*^	**2.5**^**th**^** (90% CI)**	**97.5**^**th**^** (90% CI)**	***P***_***2***_^†^	**2.5**^**th**^** (90% CI)**	**97.5**^**th**^** (90% CI)**	***P***_***3***_^‡^
RBC (× 10^12^/L)	3.73 (3.50–3.78)	5.32 (5.07–5.50)	*P*_*1*_ < 0.001	3.33 (2.98–3.38)	4.98 (4.63–5.30)	*P*_*2*_ < 0.001	3.54 (3.16–3.68)	4.98 (4.88–5.65)	*P*_*3*_ < 0.001
HGB (g/dL)	10.33 (9.25–10.77)	13.95 (13.80–14.29)	*P*_*1*_ < 0.001	9.71 (8.59–9.96)	13.17 (12.83–13.67)	*P*_*2*_ < 0.001	9.80 (8.89–10.01)	13.97 (13.80–15.00)	*P*_*3*_ < 0.001
HCT (%)	32.22 (29.82–32.83)	42.29 (41.65–43.38)	*P*_*1*_ < 0.001	30.26 (28.06–30.96)	40.07 (39.59–41.49)	*P*_*2*_ < 0.001	31.11 (28.67–31.48)	42.70 (41.80–44.39)	*P*_*3*_ = 0.003
MCV (fL)	66.13 (62.88–69.69)	95.85 (93.75–97.61)	*P*_*1*_ < 0.001	69.14 (61.94–70.88)	97.79 (96.96–98.85)	*P*_*2*_ < 0.001	69.43 (57.73–72.52)	98.40 (96.41–101.12)	*P*_*3*_ = 0.012
MCH (pg)	20.57 (19.61–22.06)	31.78 (31.17–32.65)	*P*_*1*_ < 0.001	21.51 (18.65–22.49)	32.68 (32.17–33.10)	*P*_*2*_ < 0.001	21.18 (17.30–23.00)	32.47 (32.10–33.89)	*P*_*3*_ = 0.317
MCHC (g/dL)	31.13 (30.75–31.51)	33.85 (33.75–34.11)	*P*_*1*_ = 0.403	30.99 (30.04–31.63)	34.13 (34.02–34.46)	*P*_*2*_ < 0.001	30.70 (29.96–31.10)	33.94 (33.62–34.19)	*P*_*3*_ < 0.001
RDW (%)	12.27 (11.52–12.37)	17.78 (16.95–19.60)	*P*_*1*_ < 0.001	12.69 (12.41–12.78)	16.17 (15.48–23.10)	*P*_*2*_ < 0.001	12.75 (12.24–12.89)	17.29 (16.73–20.89)	*P*_*3*_ < 0.001
RDW-SD (fL)	35.09 (33.69–36.75)	50.49 (47.25–54.69)	*P*_*1*_ < 0.001	37.97 (34.13–38.50)	48.65 (48.13–49.44)	*P*_*2*_ = 0.001	37.63 (33.25–39.38)	52.52 (50.31–55.13)	*P*_*3*_ < 0.001
WBC (× 10^9^/L)	6.33 (5.05–6.56)	15.24 (13.96–16.50)	*P*_*1*_ < 0.001	6.99 (3.52–7.34)	15.55 (14.76–16.77)	*P*_*2*_ < 0.001	6.22 (5.49–6.59)	14.14 (13.20–14.93)	*P*_*3*_ = 0.625
Neutrophil (× 10^9^/L)	3.43 (2.62–4.08)	11.70 (10.39–12.38)	*P*_*1*_ < 0.001	4.57 (1.11–4.92)	12.25 (11.15–12.98)	*P*_*2*_ < 0.001	3.99 (3.00–4.31)	10.74 (9.94–12.01)	*P*_*3*_ = 0.761
Neutrophil (%)	55.71 (44.74–57.43)	80.31 (79.14–82.79)		59.72 (31.35–63.18)	79.61 (78.40–83.64)		58.01 (49.23–59.18)	80.30 (78.58–82.72)	
Lymphocyte (× 10^9^/L)	1.22 (0.91–1.32)	3.23 (3.09–3.34)	*P*_*1*_ = 0.850	1.24 (0.90–1.33)	3.03 (2.86–3.40)	*P*_*2*_ < 0.001	1.16 (0.78–1.24)	3.06 (2.73–3.57)	*P*_*3*_ < 0.001
Lymphocyte (%)	11.83 (7.71–13.39)	34.29 (30.71–38.31)		11.94 (9.40–12.97)	29.31 (27.19–39.79)		11.59 (9.60–12.65)	31.07 (29.20–35.81)	
Monocyte (× 10^9^/L)	0.35 (0.26–0.38)	1.12 (1.01–1.25)	*P*_*1*_ < 0.001	0.38 (0.01–0.42)	1.28 (1.17–1.34)	*P*_*2*_ = 0.066	0.43 (0.02–0.46)	1.21 (1.15–1.48)	*P*_*3*_ < 0.001
Monocyte (%)	3.93 (3.39–4.51)	10.59 (10.06–11.59)		4.17 (0.13–4.52)	10.26 (9.67–11.47)		4.60 (0.23–5.15)	11.94 (11.43–15.11)	
Eosinophil (× 10^9^/L)	0.02 (0.01–0.03)	0.50 (0.39–0.92)	*P*_*1*_ < 0.001	0.04 (0.00–0.05)	0.50 (0.44–0.86)	*P*_*2*_ < 0.001	0.03 (0.02–0.04)	0.39 (0.35–0.54)	*P*_*3*_ = 0.152
Eosinophil (%)	0.22 (0.08–0.28)	4.79 (4.42–6.55)		0.34 (0.05–0.49)	4.64 (4.12–7.86)		0.33 (0.16–0.37)	3.74 (3.39–4.91)	
Basophil (× 10^9^/L)	0.01 (0.01–0.01)	0.09 (0.08–0.13)	*P*_*1*_ = 0.769	0.01 (0.00–0.01)	0.09 (0.08–0.11)	*P*_*2*_ = 0.993	0.01 (0.01–0.01)	0.09 (0.08–0.15)	*P*_*3*_ = 0.708
Basophil (%)	0.10 (0.07–0.13)	0.92 (0.80–1.22)		0.10 (0.00–0.12)	0.76 (0.69–0.92)		0.12 (0.06–0.13)	0.93 (0.79–1.61)	
Platelet (× 10^9^/L)	169.66 (157.10–192.40)	413.88 (389.50–508.40)	*P*_*1*_ = 0.007	172.34 (85.40–182.60)	372.19 (359.10–426.00)	*P*_*2*_ = 0.157	151.30 (117.80–160.50)	417.14 (400.40–491.30)	*P*_*3*_ < 0.001
MPV (fL)	6.65 (6.39–6.74)	9.85 (9.67–9.99)	*P*_*1*_ = 0.139	6.48 (6.11–6.63)	9.70 (9.36–9.92)	*P*_*2*_ < 0.001	6.69 (6.29–6.96)	10.39 (9.98–11.19)	*P*_*3*_ < 0.001

^*^P values for First trimester vs Second trimester

^†^*P* values for Second trimester vs Third trimester

^‡^*P* values for First trimester vs Third trimester

**Table 3:** Reference intervals for coagulation tests

Parameter	**First trimester (*n* = 288)**			**Second trimester (*n* = 269)**			**Third Trimester (*n* = 279)**		
	**2.5**^**th**^** (90% CI)**	**97.5**^**th**^** (90% CI)**	***P***_1_^*^	**2.5**^**th**^** (90% CI)**	**97.5**^**th**^** (90% CI)**	***P***_***2***_^†^	**2.5**^**th**^** (90% CI)**	**97.5**^**th**^** (90% CI)**	***P***_***3***_^‡^
PT (seconds)	10.30 (10.10–10.30)	12.88 (12.40–13.20)	*P*_*1*_ < 0.001	9.80 (9.60–9.90)	11.60 (11.50–11.90)	*P*_*2*_ < 0.001	9.60 (9.30–9.70)	11.40 (11.20–11.90)	*P*_*3*_ < 0.001
APTT (seconds)	25.40 (22.10–25.60)	35.46 (34.30–36.60)	*P*_*1*_ < 0.001	24.05 (22.40–24.30)	33.23 (32.60–35.20)	*P*_*2*_ < 0.001	23.40 (21.20–23.80)	31.80 (31.40–33.60)	*P*_*3*_ < 0.001
Fibrinogen (mg/dL)	280.28 (181.40–304.85)	559.00 (531.93–593.35)	*P*_*1*_ < 0.001	347.75 (314.23–366.02)	593.35 (561.26–616.45)	*P*_*2*_ < 0.001	330.28 (261.71–366.83)	628.56 (604.72–667.32)	*P*_*3*_ < 0.001

^*^P values for First trimester vs Second trimester

^*†*^*P* values for Second trimester vs Third trimester

^*‡*^*P* values for First trimester vs Third trimester

**Table 4:** Verification for transference of reference interval

**Parameter**	**Number of accepted samples per 20 samples (First trimester)**	**First trimester 2.5**^**th**^**–97.5**^**th**^	**Number of accepted samples per 20 samples (Second trimester)**	**Second trimester 2.5**^**th**^**–97.5**^**th**^	**Number of accepted samples per 20 samples (Third trimester)**	**Third Trimester 2.5**^**th**^**–97.5**^**th**^
**Aiwei Li et al. **[7]						
**RBC (× 10**^**12**^**/L)**	20/20	3.07–5.07	20/20	2.85–4.59	19/20	2.75–4.64
**HGB (g/dL)**	20/20	11.0–14.7	20/20	8.80–13.60	20/20	8.40–14.10
**HCT (%)**	20/20	33.0–43.00	20/20	27.00–40.00	19/20	26.00–42.00
**MCV (fL)**	20/20	76.80–95.20	20/20	78.3–99.7	20/20	78.7–101.7
**MCH (pg)**	20/20	24.60–32.70	14/20	24.60–30.00	20/20	25.1–34.6
**MCHC (g/dL)**	20/20	32.00–35.50	19/20	31.90–35.10	20/20	31.5–34.8
**RDW (%)**	20/20	11.90–16.80	20/20	12.30–17.20	20/20	12.3–19.8
**WBC (× 10**^**9**^**/L)**	19/20	4.68–12.87	20/20	5.97–16.78	20/20	5.53–19.56
**Neutrophil (× 10**^**9**^**/L)**	19/20	2.72–9.92	20/20	4.16–14.11	19/20	3.73–17.24
**Lymphocyte (× 10**^**9**^**/L)**	20/20	1.11–3.05	20/20	0.86–2.88	20/20	0.70–2.60
**Monocyte (× 10**^**9**^**/L)**	14/20	0.20–0.66	20/20	0.22–0.98	16/20	0.26–1.10
**Eosinophil (× 10**^**9**^**/L)**	20/20	0.01–0.31	20/20	0.00–0.35	18/20	0.00–0.23
**Basophil (× 10**^**9**^**/L)**	19/20	0.00–0.05	20/20	0.00–0.08	16/20	0.00–0.05
**Platelet (× 10**^**9**^**/L)**	19/20	148–352	19/20	111–346	16/20	80–309
**MPV (fL)**	7/20	8.5–11.9	19/20	7.0–11.8	19/20	7.0–12.9
**C. Cui et al. **[2]						
**PT (seconds)**	20/20	9.7–12.5	20/20	8.5–13.2	20/20	8.6–12.4
**APTT (seconds)**	19/20	26.4–41.9	20/20	24.4–35.8	20/20	25.6–34.9
**Fibrinogen (mg/dL)**	18/20	238–444	20/20	240–597	18/20	279–591

## Discussion

**Fig. 1:**
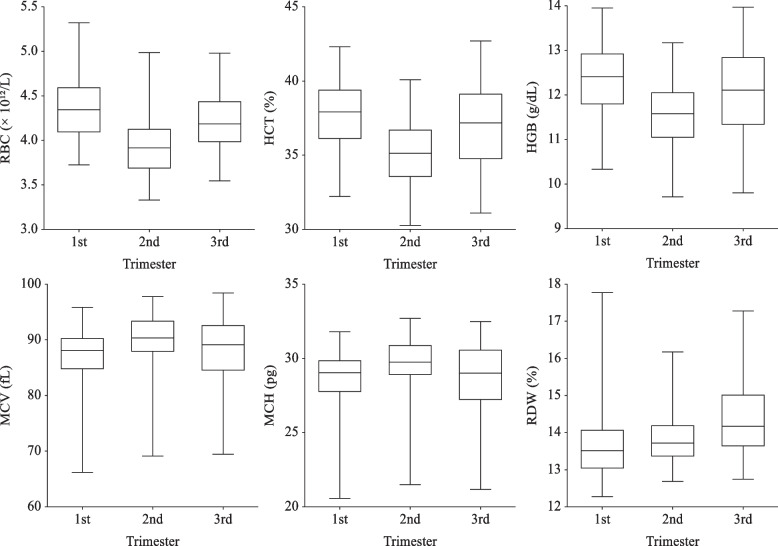
RBC, HCT, HGB, MCV, MCH, and RDW during pregnancy. The middle line reflects the median, and the crossbars represents the 2.5^th^ and 97.5^th^ percentiles

During pregnancy, the mother has an increase in blood volume, up to 50% increase. This contributes to slightly decreased red blood cell indices, also known as dilutional anemia. However, red blood cell production also increases by 25–30% until term gestation [1]. In our study, RBC, HCT, and HGB (Fig. 1) started to decline in the second trimester but then rise again in the third trimester, which is explained by the physiology adaptation in pregnancy. The 2.5^th^ percentiles of HGB (g/dL) in our study are 10.33, 9.71, 9.80 in first, second and third trimester, respectively. Nevertheless, according to the definition of anemia recommended by Centers for Disease Control and Prevention (CDC) [8], HGB levels below 11 g/dL, 10.5 g/dL, 11 g/dL in first, second and third trimester, respectively, are considered to be anemic. 11% of patients were classified as anemia if we use CDC criteria despite taking taking iron supplements. Several studies including Aiwei Li et al. and Yi Jin et al. [4, 7] also found that the 2.5^th^ percentiles were below the diagnostic cutoff for anemia. An explanation for this phenomenon may be due to the use of 5^th^ percentile of data aggregated from four European studies of healthy iron-supplemented pregnant women to establish the cutoff [9]. Therefore, CDC conventional diagnostic criteria for anemia may not be approriate for Vietnamese pregnant women and we suggest the use of 2.5^th^ percentile as a cutoff for anemia diagnosis.

**Fig. 2:**
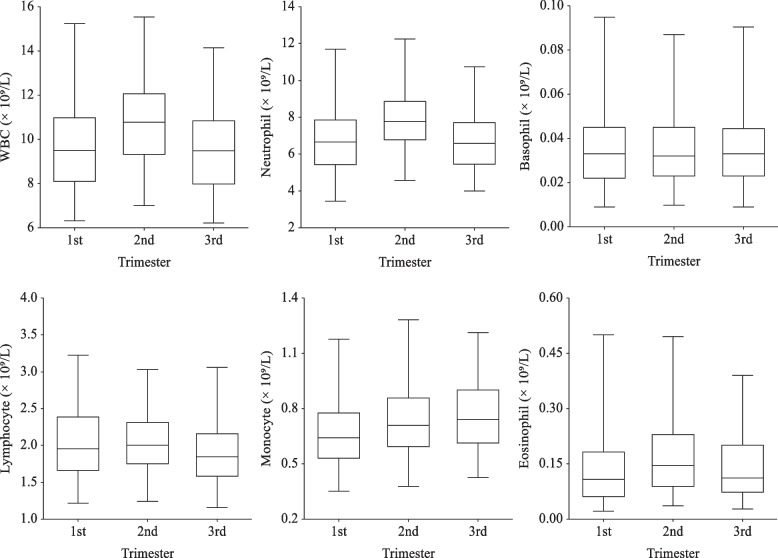
WBC, Neutrophil, Basophil, Lymphocyte, Monocyte, and Eosinophil during pregnancy. The middle line reflects the median, and the crossbars represents the 2.5^th^ and 97.5^th^ percentiles

In addition, Torgersen CKL and Curran CA [1] reported white blood cell count increases in the beginning of the first trimester and then remains stable in the second and third trimester. In white blood cell count, neutrophils contributes the most to the rise, followed by eosinophils. In our study, white blood cell count from all trimesters ranges from nearly 6 × 10^9^/L to roughly 15 × 10^9^/L, with the number of white blood cell count increases slightly in second trimester, compared to the first trimester, but decreases in the third trimester (Fig. 2). Thus, the difference between the third trimester and first trimester shows no statistical significance.

**Fig. 3:**
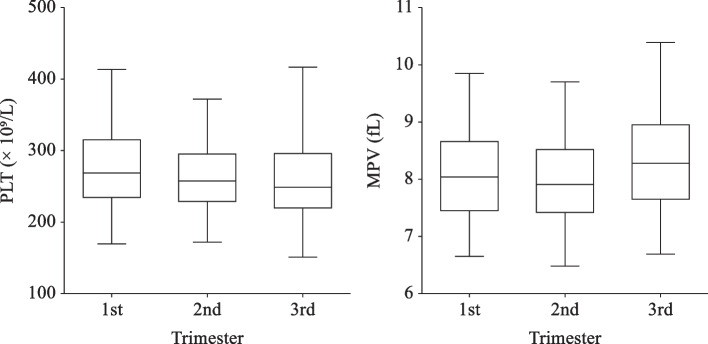
PLT and MPV during pregnancy. The middle line reflects the median, and the crossbars represents the 2.5^th^ and 97.5^th^ percentiles

Because of dilutional effect in pregnancy, platelet count also suffers from the same decrease as red blood cell count. Figure 3 shows the platelet count are roughly equaled. Only 9 (1%) women in our study suffered from thrombocytopenia but they did not show any signs or symtomps. Nonetheless, the 2.5^th^ percentile of platelet count in each trimester is well above the 150 × 10^9^/L cutoff for thrombocytopenia [10]. Hence, platelet count in pregnant women is almost the same as non-pregnant women.

**Fig. 4:**
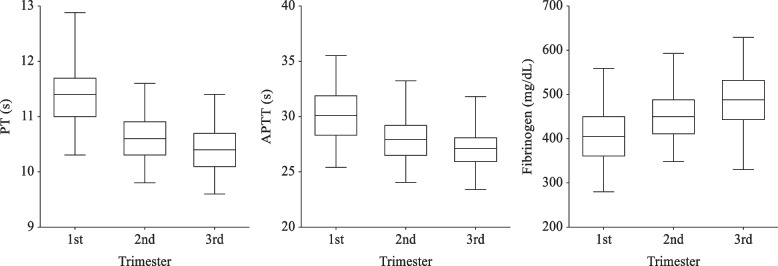
PT, APTT, and Fibrinogen during pregnancy. The middle line reflects the median, and the crossbars represents the 2.5^th^ and 97.5^th^ percentiles

PT, APTT, Fibrinogen reference intervals are presented in Table 3. Figure 4 also shows that PT and APTT get shorter in every trimester while fibrinogen level shows an ascending trend as gestational age increase. This can be explained by the hypercoagulable state of pregnant women. Our results coincide with the results of C. Cui et al. [2], which also used the ACL TOP coagulation analyzer. Moreover, the first trimester medians of PT (s), APTT (s) in our study are 11.35 and 30.1, respectively, which are close to non-pregnancy medians in C. Cui’s study (11.0, 32.8). A meta-analysis study by Mina Abbassi-Ghanavati et al. [11] also found a similar hypercoagulation trend. The difference between each trimester reference interval was statistically significant.

Apart from establishing new reference intervals, we performed a verification study which is described in Table 4. Before verifying the transferability, we randomly selected 20 persons from our study and perform a Tukey outlier test until there were no outliers for all tests from the complete blood count panel or coagulation tests. After we had successfully chosen 20 persons with no outliers in the data, we tested if there were more than two results that fell outside of the targeted reference interval. If less than or equal to two results fall outside, the reference interval is verified. This procedure is recommended by CLSI EP28-A3c. Overall, in complete blood count verification, we used data from Aiwei Li’s study [7], which used Sysmex XE-2100 hematology analyzer. Most of the parameters were verified except for platelet parameter in third trimester and MPV parameter in first trimester and monocyte in first and third trimesters. In coagulation test verification, we used data from C. Cui’s study [2], which used ACP TOP coagulation analyzer. All coagulation parameters fell inside the limits, proving that the verification process can be successfully achieved if the chosen study had the same equipment as the laboratory’s. Another point to make is that the patient selection step for verification can be cumbersome because we must find another patient to replace the one that has outliers in her test results.

## Conclusions

Reference intervals are important tools to aid clinicians in deciding their next step in treatment. Thus, it is important that laboratories use appropriate reference intervals via verifying or establishing new ones. In our study, the trimester-specific reference intervals were derived from random and independent women. In order to determine the real change in women during pregnancy, data throughout three trimesters should be taken from one participant. Therefore, our study’s results may be affected by the biologic variations between subjects. Despite that, by obtaining data from a large sample, we hope to minimize that effect and bring clinicians a useful tool to evaluate and other laboratories our own target population reference intervals.

## Data Availability

The datasets used and/or analyzed during the current study are available from the corresponding author on reasonable request.

## Abbreviations

APTT: Activated Partial Thromboplastin Time
CI: Confidence interval
CLSI: Clinical and Laboratory Standards Institute
FIB: Fibrinogen
HCT: Hematocrit
HGB: Hemoglobin
MCH: Mean corpuscular hemoglobin
MCHC: Mean corpuscular hemoglobin concentration
MPV: Mean corpuscular volume
PLT: Platelet
PT: Prothrombin time
RBC: Red blood cell
RDW: RBC distribution width
